# SCAR-Net-assisted ultrasound diagnosis of postoperative scars and recurrent lesions in breast cancer

**DOI:** 10.1016/j.isci.2026.115550

**Published:** 2026-04-01

**Authors:** Na Feng, Shanshan Zhao, Zhikai Lei, Dan Yi, Jincao Yao, Xiao Zhang, Xiangyang Li, Weijie Zou, Jian Zhang, Liyu Chen, Chen Yang, Dong Xu, Yong Wu

**Affiliations:** 1 Department of Diagnostic Ultrasound Imaging & Interventional Therapy, Zhejiang Cancer Hospital, Hangzhou Institute of Medicine (HIM), Chinese Academy of Sciences, Hangzhou, China; 2 Department of Ultrasound, Shaoxing People’s Hospital (Zhejiang University Shaoxing Hospital), Shaoxing, China; 3 The First Affiliated Hospital of Zhejiang Chinese Medical University (Zhejiang Provincial Hospital of Chinese Medicine), Hangzhou, China; 4 Interventional Medicine and Engineering Research Center, Hangzhou Institute of Medicine (HIM), Chinese Academy of Sciences, Hangzhou, China; 5 Zhejiang Provincial Research Center for Innovative Technology and Equipment in Interventional Oncology, Zhejiang Cancer Hospital, Hangzhou, China; 6 The First People’s Hospital of Lin’an District, Hangzhou, China; 7 Wenzhou Medical University, Wenzhou, China; 8 Department of Medical Engineering, Zhejiang Cancer Hospital, Hangzhou Institute of Medicine (HIM), Chinese Academy of Sciences, Hangzhou, China

**Keywords:** oncology, medical imaging, artificial intelligence applications

## Abstract

Postoperative differentiation between scar tissue and recurrent lesions in patients with breast cancer presents a significant diagnostic challenge. This study introduces SCAR-Net, a deep learning model specifically designed for ultrasound-based discrimination between these similar-appearing tissues. Using 34,376 ultrasound images from 5,710 patients across four hospitals, we developed a model incorporating scar-recurrence feature enhancer and boundary-sensitive attention network modules. In multicenter validation, SCAR-Net significantly improved radiologists' diagnostic performance, increasing AUC from 0.810–0.824 to 0.939–0.942, sensitivity from 0.775–0.782 to 0.934–0.941, and specificity from 0.839–0.872 to 0.935–0.950 (all *p* < 0.001). The model also demonstrated superior segmentation accuracy with Dice coefficients of 0.918–0.925 compared to traditional methods (0.795–0.811). These results suggest SCAR-Net could serve as a valuable auxiliary tool for improving early recurrence detection in postoperative breast cancer follow-up, potentially reducing unnecessary biopsies and enabling more timely interventions.

## Highlights

• Introduces an SCAR-Net model for differentiating scars from recurrence in breast ultrasound
• Enhances radiologists’ diagnostic accuracy regardless of experience level
• Provides a standardized tool for post-operative breast cancer follow-up

## Graphical abstract

**Figure:**
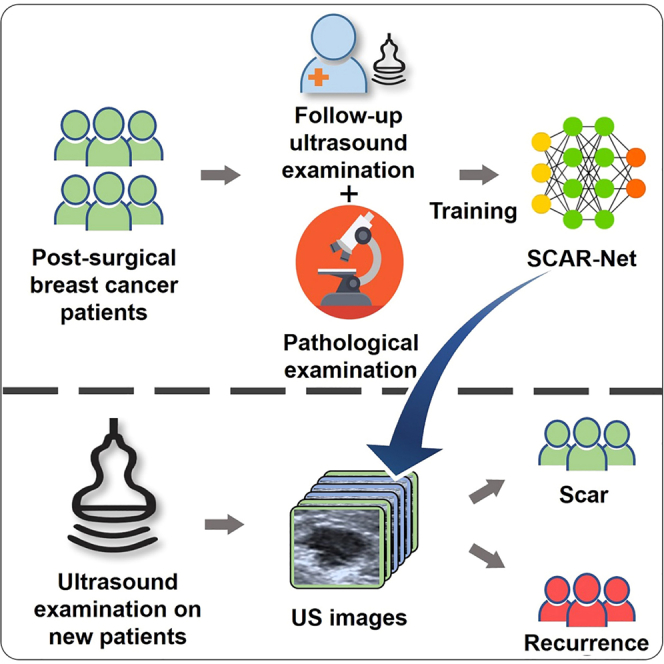

## Introduction

Breast cancer is the most common malignant tumor among women worldwide.^1^ It accounts for approximately 24.2% of all female cancers, with an incidence rising annually.^2^^,^^3^ Statistics show that around 85–90% of patients with early-stage breast cancer undergo surgical treatment, with breast-conserving surgery accounting for 60–70% and mastectomy for 30–40%.^4^^,^^5^^,^^6^^,^^7^ Among these surgically treated patients, approximately 3–10% experience local recurrence within 5 years.^2^^,^^4^^,^^5^ Timely detection of these local recurrences is crucial for determining subsequent treatment plans and improving patient survival rates. However, following surgical treatment, the healing process in the traumatized area triggers complex biological responses and tissue reconstruction, ultimately forming scar tissue.^6^^,^^7^ These scar tissues closely resemble breast cancer recurrent lesions in clinical presentation, which poses significant challenges for postoperative follow-up and timely diagnosis of recurrence.^8^^,^^9^^,^^10^

Ultrasound examination has become the preferred imaging method for postoperative breast cancer follow-up due to its non-invasive, convenient, economical, and radiation-free advantages.^11^^,^^12^^,^^13^ Generally, post-surgical scars and breast cancer recurrence commonly appear as hypoechoic areas on ultrasound examination. Conventionally, scar tissue is thought to present with relatively regular morphology, well-defined borders, and homogeneous internal echoes, while recurrent lesions tend to exhibit irregular margins, heterogeneous internal echoes, accompanied by posterior acoustic shadowing and fine spiculations on ultrasound.^14^ However, these features are relative descriptions rather than absolute criteria. In clinical practice, early recurrent lesions may present with regular morphology and homogeneous echoes similar to scars, while post-surgical scars during tissue remodeling may also display “malignant features” such as irregular borders, heterogeneous internal echoes, or posterior acoustic shadowing. This overlap of features creates diagnostic dilemmas even for experienced radiologists.^15^^,^^16^ This creates difficulties in precisely differentiating between scar tissue and recurrent breast cancer lesions, resulting in increased diagnostic uncertainty, unnecessary biopsy rates, and possible delays in recurrence diagnosis.^9^^,^^12^

In recent years, artificial intelligence (AI) technology, especially deep learning (DL), has been increasingly applied in medical imaging.^17^^,^^18^^,^^19^^,^^20^^,^^21^ AI-assisted systems have achieved performance levels comparable to or exceeding those of professional radiologists in detecting primary breast tumors and differentiating between benign and malignant lesions on ultrasound.^22^^,^^23^ In postoperative breast cancer surveillance, conventional imaging modalities remain the mainstay of clinical assessment. Color Doppler combined with contrast-enhanced ultrasound can reveal the vascular distribution patterns of recurrent lesions, increasing diagnostic sensitivity from 64% to 86%,^24^ while MRI demonstrates sensitivity and specificity of 90% and 91.6%, respectively, in evaluating suspicious areas after breast-conserving surgery.^25^ Yet postoperative structural distortion, heterogeneous neovascularization, and contrast-agent limitations make these modalities prone to misinterpretation when distinguishing postoperative scar tissue from early recurrence. Meanwhile, several studies have attempted to predict recurrence risk or localization using clinical and imaging features. For instance, machine learning models have been applied to predict local recurrence and distant metastasis, achieving AUCs of 0.75–0.69 across different tissue regions,^26^ and an XGBoost-based approach attained an AUC of 0.97 in identifying recurrence at the postoperative tumor bed.^27^ Nevertheless, these studies were mostly single-center and small-scale, relying on static features with limited generalizability. In contrast, DL has achieved remarkable progress in the classification and segmentation of breast lesions. However, despite rapid advancements in AI for primary breast tumor diagnosis, research on ultrasound differentiation between scars and recurrent lesions in postoperative breast cancer contexts remains relatively scarce, and existing models cannot be directly applied to this specific clinical problem.^28^^,^^29^^,^^30^^,^^31^^,^^32^

To address this clinical challenge, we innovatively propose SCAR-Net, an AI-assisted diagnostic model specifically designed for ultrasound differentiation between postoperative scars and recurrent lesions in breast cancer. Based on the characteristics of scars and recurrent lesions, this model introduces two innovative modules: the scar-recurrence feature enhancer (SRFE) to improve texture feature extraction capabilities for scars and recurrent lesions and the boundary-sensitive attention network (BSAN) to enhance segmentation accuracy. We collected 34,376 postoperative follow-up ultrasound images from 5,710 patients across four medical centers and established internal validation and external test sets to verify the model’s generalizability.

To our knowledge, SCAR-Net is the first DL model based on ultrasound specifically designed for differentiating postoperative scars from recurrent lesions in breast cancer. Additionally, this study represents one of the largest multicenter AI-assisted diagnostic studies on postoperative breast cancer scars and recurrent lesions in the field of ultrasound imaging. We compared the diagnostic performance of ultrasound radiologists with varying experience levels with and without AI assistance and explored the practical benefits of AI in improving diagnostic accuracy. This SCAR-Net-based research provides technical support and clinical decision-making bases for follow-up management and precise diagnosis of patients with postoperative breast cancer.

## Results

### Study design and datasets

**Figure 1:**
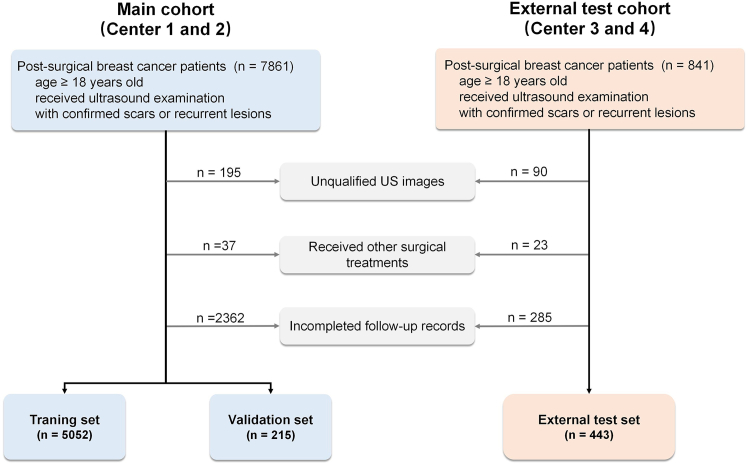
Flowchart of data inclusion process for the main and external validation cohorts Centers 1 to 4 represent Zhejiang Cancer Hospital, Shaoxing People’s Hospital, Zhejiang Provincial Hospital of Chinese Medicine, and the First People’s Hospital of Lin’an, respectively.

Figure 1 shows the patient enrollment flowcharts for the main and external test cohorts, as well as the methodology for establishing the training, validation, and external test sets. Table 1 provides more detailed distribution information for the datasets. The detailed inclusion criteria were (1) female patients with breast cancer aged ≥18 years who underwent surgical treatment; (2) patients who received ultrasound examinations during postoperative follow-up, with recorded ultrasound images; and (3) recurrent lesions confirmed by pathological results and scars either confirmed by pathological results or verified through more than one year of follow-up without progression. According to these enrollment criteria, we retrospectively collected 71,923 ultrasound images from a total of 8,702 patients across four multicenter hospitals. The exclusion criteria were (1) poor-quality ultrasound images or ultrasound images where the lesion location was unrelated to the surgical area, (2) patients who underwent breast reconstruction surgery or other breast-related surgeries, and (3) patients with interrupted follow-up or incomplete follow-up data. After screening based on these criteria, we ultimately retained 6,165 lesions and 34,376 ultrasound images from 5,710 patients with postoperative breast cancer. Detailed data collection information for each center is provided in Table S1.

**Table 1:** Baseline characteristics

Category	Training set	Validation set	External test set
Number of patients	5052	215	443
Number of nodules	5453	236	476
Number of images	31047	1371	1958
Age, mean (SD)	47.7 (8.6)	47.2 (9.1)	48.6(9.7)
Size, mm (SD)	8.6 (4.1)	8.7(4.7)	8.4 (4.3)
Lesions			
Scar	4910	161	385
Recurrence	543	75	91

SD: Standard deviation.

### The AI model

**Figure 2:**
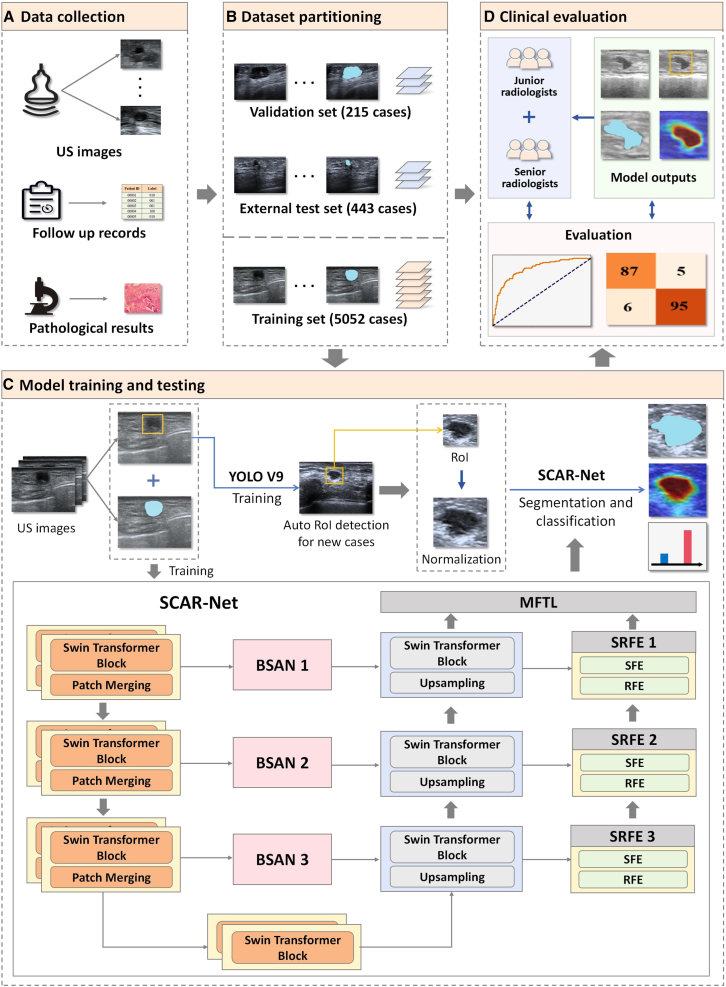
Overall design of models and experiments (A) Multicenter collection of ultrasound images of scars and recurrent lesions, follow-up records, and pathological results. (B) Division of multicenter data into the training set, validation set, and external test set. (C) Construction of the two-stage pipeline: YOLO V9 rapidly detects ROIs containing potential lesions, which are then input into SCAR-Net for segmentation and classification. (D) Evaluation of SCAR-Net model’s diagnostic performance and testing of SCAR-Net model’s diagnostic assistance effect for radiologists with varying years of experience. Detailed module design of SCAR-Net and its subnetworks can be found in Tables S2–S5.

SCAR-Net is a DL network model specifically designed for computer-aided diagnosis of postoperative breast cancer scars and recurrences on ultrasound, integrating both segmentation and classification functions for post-surgical breast cancer scars and recurrent lesions. Figure 2 shows the overall design of the model and experiments. During preprocessing, the YOLO V9 model was used to rapidly detect the locations of scars and recurrent lesions, and these scar or recurrent lesion ROIs were subsequently input into the SCAR-Net model for segmentation and identification. SCAR-Net adopts Swin-Unet as its backbone and introduces two innovative attention modules: the BSAN and SRFE. BSAN enhances boundary regions of scars and recurrent lesions by focusing on areas with significant gradient changes, while SRFE enhances specific texture and morphological features while suppressing surrounding tissue interference. A multi-feature transformer layer (MFTL) aggregates features to complete the final diagnostic task. Detailed designs of SCAR-Net and its BSAN, SRFE, and MFTL subnetworks are referenced in Tables S2–S5. Additionally, a comprehensive network architecture diagram is provided in Figure S3 for visual illustration.

### Performance in assisting radiologists

**Figure 3:**
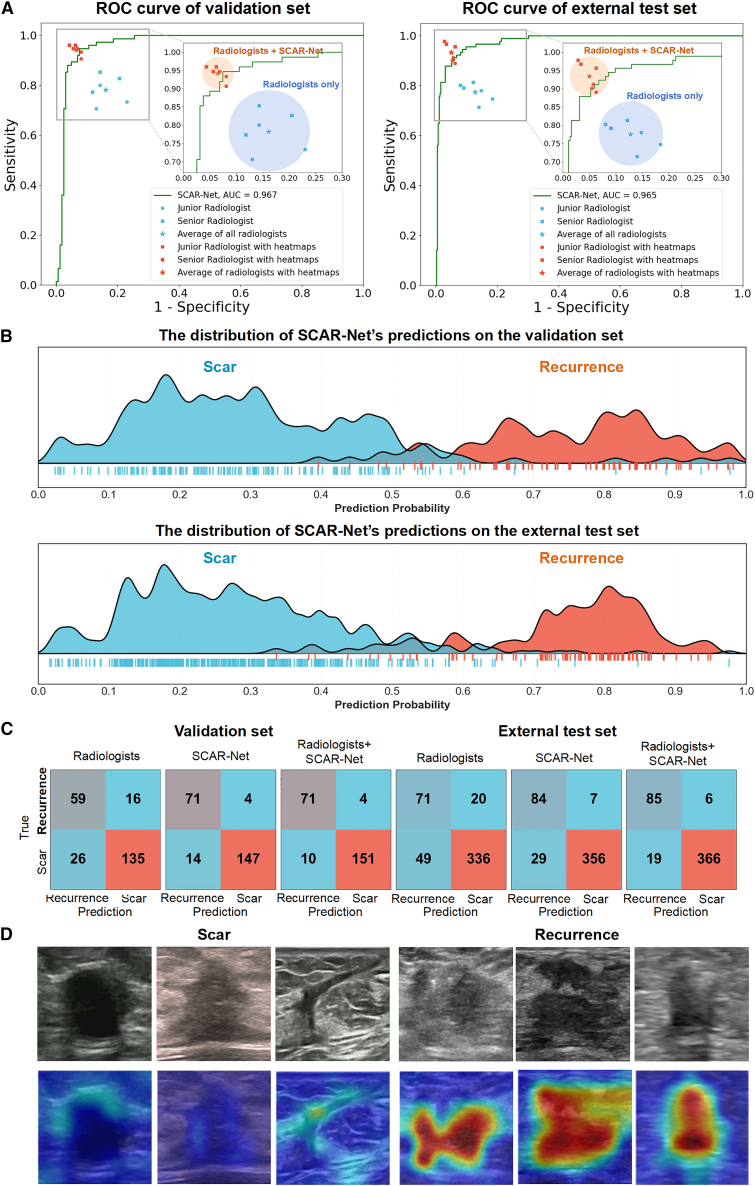
Diagnostic performance and visualization of SCAR-Net for differentiating scars and recurrent lesions (A) Left: ROC curve of SCAR-Net on the validation set and comparison of diagnostic results between radiologists’ independent diagnosis and diagnosis assisted by SCAR-Net. Right: ROC curve on the external test set and comparison of diagnostic results between radiologists’ independent diagnosis and diagnosis assisted by SCAR-Net. The key areas were magnified, and circles were drawn centered at the mean point with a radius equal to the maximum eccentric value, which outlined the coverage range of diagnostic results by radiologists under different conditions. (B) Distribution of prediction probability values from SCAR-Net in the validation set and external test set, shows clustering of scars and recurrent lesions. (C) Confusion matrices illustrate true versus predicted classes. (D) First row: original ultrasound images of scars and recurrent lesions; second row: feature heat maps output by the SCAR-Net model.

SCAR-Net demonstrated excellent performance in the segmentation and diagnosis of postoperative scars and recurrent lesions on breast cancer ultrasound images. Figure 3 comprehensively displays the model’s diagnostic capabilities. From the ROC curve analysis (Figure 3A), SCAR-Net exhibited steep curve patterns on both the validation and external test sets, with AUC values reaching 0.967 and 0.965, respectively, exceeding the independent diagnostic level of radiologists. The prediction probability distribution graph (Figure 3B) shows that the model had clear discriminative power, with small overlapping areas in the probability distributions of scars and recurrent lesions, indicating clustering phenomena and reflecting the model’s good ability to differentiate between scars and recurrent lesions. To further illustrate case-level performance and class balance, confusion matrices were added (Figure 3C), comparing independent radiologists, SCAR-Net alone, and AI-assisted readings across both cohorts. The matrices demonstrate that SCAR-Net substantially reduced false negatives for recurrence while maintaining high specificity for scars. In addition to discriminatory performance, SCAR-Net demonstrated well-calibrated probability estimates across both cohorts, with low Brier scores (0.107 and 0.096) and modest underconfidence indicated by ECE values (∼0.21; Figure S4).

During the AI-assisted reading phase, radiologists reviewed each case via a customized workstation interface seamlessly integrated into the existing picture archiving and communication systems (PACS) workflow. The interface presented four key components to facilitate comprehensive assessment: (1) the original ultrasound images, (2) SCAR-Net-generated lesion contours that automatically delineated the region of interest with pixel-level precision, (3) quantitative recurrence-risk probabilities expressed as percentage scores with confidence intervals, and (4) attention heatmaps that visually highlighted the most discriminative morphological features contributing to the risk prediction. This multi-modal visual guidance enabled radiologists to efficiently localize suspicious lesions, cross-validate AI predictions against their clinical expertise, and focus their attention on critical imaging features that might otherwise be overlooked during routine examination.

Table 2 presents the classification performance of different models, with SCAR-Net achieving an AUC of 0.967 (95% CI: 0.942–0.988) on the validation set, significantly outperforming other DL methods such as DeepLabV3+ (0.810), Unet (0.815), Unet++ (0.826), and Swin-Unet (0.855). Similarly, on the external test set, SCAR-Net’s AUC was 0.965 (95% CI: 0.958–0.967), also significantly superior to other comparative models. For the identification of recurrent lesions, SCAR-Net achieved sensitivities of 0.947 and 0.923 in the validation and external test sets, respectively, substantially higher than other models, while maintaining high specificities of 0.913 and 0.925.

**Table 2:** Comparison of the classification capabilities of various deep learning methods

Methods	AUC (95% CI)	TPR (95% CI)	TNR (95% CI)	PPV (95% CI)	NPV (95% CI)	ACC (95% CI)	F1
Validation set							
DeepLabV3+	0.810 (0.743, 0.867)	0.760 (0.658, 0.846)	0.783 (0.720, 0.844)	0.620 (0.523, 0.724)	0.875 (0.819, 0.925)	0.775 (0.725, 0.831)	0.683
Unet	0.815 (0.757, 0.872)	0.760 (0.662, 0.848)	0.807 (0.745, 0.864)	0.648 (0.545, 0.745)	0.878 (0.822, 0.929)	0.792 (0.742, 0.843)	0.697
Unet++	0.826 (0.763, 0.878)	0.773 (0.667, 0.862)	0.795 (0.728, 0.859)	0.637 (0.543, 0.727)	0.883 (0.828, 0.932)	0.788 (0.733, 0.839)	0.699
Swin-Unet	0.855 (0.802, 0.906)	0.787 (0.688, 0.871)	0.832 (0.771, 0.889)	0.686 (0.578, 0.787)	0.893 (0.845, 0.940)	0.818 (0.767, 0.864)	0.733
SCAR-Net (BSAN only)	0.887 (0.838, 0.934)	0.840 (0.754, 0.922)	0.845 (0.785, 0.902)	0.716 (0.610, 0.812)	0.919 (0.871, 0.959)	0.843 (0.801, 0.890)	0.773
SCAR-Net (SRFE only)	0.863 (0.803, 0.913)	0.800 (0.705, 0.895)	0.851 (0.791, 0.902)	0.714 (0.614, 0.802)	0.901 (0.852, 0.945)	0.835 (0.784, 0.881)	0.755
SCAR-Net	0.967 (0.942, 0.988)	0.947 (0.889, 0.988)	0.913 (0.865, 0.955)	0.835 (0.757, 0.908)	0.974 (0.943, 0.994)	0.924 (0.886, 0.953)	0.887
External test set							
DeepLabV3+	0.787 (0.726, 0.843)	0.736 (0.644, 0.821)	0.816 (0.776, 0.856)	0.486 (0.394, 0.571)	0.929 (0.902, 0.955)	0.800 (0.765, 0.836)	0.585
Unet	0.804 (0.745, 0.860)	0.725 (0.632, 0.810)	0.839 (0.802, 0.876)	0.516 (0.432, 0.600)	0.928 (0.896, 0.953)	0.817 (0.782, 0.851)	0.603
Unet++	0.830 (0.774, 0.879)	0.747 (0.660, 0.831)	0.855 (0.821, 0.890)	0.548 (0.462, 0.632)	0.935 (0.909, 0.959)	0.834 (0.800, 0.866)	0.633
Swin-Unet	0.845 (0.791, 0.897)	0.802 (0.719, 0.885)	0.844 (0.806, 0.877)	0.549 (0.461, 0.635)	0.948 (0.926, 0.970)	0.836 (0.803, 0.866)	0.652
SCAR-Net (BSAN only)	0.872 (0.826, 0.916)	0.835 (0.750, 0.908)	0.844 (0.806, 0.879)	0.559 (0.479, 0.643)	0.956 (0.933, 0.976)	0.842 (0.809, 0.874)	0.671
SCAR-Net (SRFE only)	0.854 (0.801, 0.901)	0.813 (0.731, 0.887)	0.834 (0.797, 0.871)	0.536 (0.454, 0.616)	0.950 (0.925, 0.971)	0.830 (0.796, 0.861)	0.646
SCAR-Net	0.965 (0.958, 0.967)	0.923 (0.850,0.962)	0.925 (0.894,0.947)	0.825 (0.737,0.888)	0.971 (0.949,0.984)	0.941 (0.916,0.959)	0.851

Furthermore, Table 2 presents ablation experiments using the BSAN module alone and the SRFE module alone to verify the effectiveness of each module in SCAR-Net. On the validation set, compared to the baseline model Swin-Unet (AUC = 0.855), using only the BSAN module increased the AUC to 0.887 (an improvement of 3.2%), while using only the SRFE module merely increased the AUC to 0.863 (an improvement of 0.8%), indicating that the improvement effect of a single module is limited. However, the complete SCAR-Net model, integrating both modules, significantly increased the AUC to 0.967, representing an 11.2% improvement over the baseline. The external test set showed a consistent trend: compared to Swin-Unet (AUC = 0.845), using only the BSAN module increased the AUC to 0.872 (an improvement of 2.7%), while using only the SRFE module merely increased the AUC to 0.854 (an improvement of 0.9%); whereas the complete SCAR-Net model achieved an AUC of 0.965, representing a 12.0% improvement over the baseline. These results confirm that optimizing only the segmentation module, although capable of improving target region localization, has a limited impact on classification performance; simultaneously enhancing feature extraction and boundary sensitivity can produce synergistic effects, significantly improving the accuracy of distinguishing between scars and recurrent lesions.

Tables 3 and 4 compare the diagnostic results of the radiologists’ independent diagnoses and their diagnoses with assistance from various models. As shown in Table 3, in the validation set, the average AUC of the radiologists’ independent diagnosis was 0.810 (95% CI: 0.788–0.831), which increased to 0.939 (95% CI: 0.924–0.951, *p* < 0.001) with SCAR-Net assistance. Notably, junior radiologists’ diagnostic ability improved from 0.789 to 0.926 with AI assistance, nearly reaching the level of senior radiologists with AI assistance (0.951). For sensitivity to recurrent lesions, AI assistance significantly improved all radiologists’ performance from 0.782 to 0.941. Similar improvements were observed in the external test set, where the radiologists’ average AUC for independent diagnosis was 0.824 (95% CI: 0.805–0.842), increasing to 0.942 (95% CI: 0.931–0.953, *p* < 0.001) with SCAR-Net assistance. The diagnostic performance of each radiologist under each method of assistance in the validation and external test sets can be found in Tables S6 and S7, respectively.

**Table 3:** Comparison of assisted diagnostic results in the validation set

Modalities	AUC (95% CI)	TPR (95% CI)	TNR (95% CI)	PPV (95% CI)	NPV (95% CI)	ACC (95% CI)	F1
Radiologists only							
All	0.810 (0.788, 0.831)	0.782 (0.742,0.818)	0.839 (0.814,0.860)	0.693 (0.651,0.731)	0.892 (0.870,0.911)	0.821 (0.800,0.840)	0.735
Junior	0.789 (0.755, 0.823)	0.747 (0.686,0.799)	0.832 (0.796,0.863)	0.675 (0.614,0.730)	0.876 (0.843,0.903)	0.805 (0.774,0.833)	0.709
Senior	0.831 (0.799, 0.858)	0.818 (0.762,0.863)	0.845 (0.810,0.874)	0.710 (0.652,0.762)	0.909 (0.879,0.932)	0.836 (0.807,0.862)	0.760
Radiologists with Swin-Unet							
All	0.838 (0.818, 0.858)	0.844 (0.808,0.875)	0.831 (0.806,0.854)	0.700 (0.660,0.737)	0.920 (0.900,0.936)	0.835 (0.815,0.854)	0.765
Junior	0.821 (0.790, 0.849)	0.818 (0.762,0.863)	0.824 (0.788,0.855)	0.684 (0.626,0.737)	0.907 (0.876,0.930)	0.822 (0.792,0.848)	0.745
Senior	0.855 (0.827, 0.884)	0.871 (0.821,0.909)	0.839 (0.803,0.869)	0.715 (0.659,0.766)	0.933 (0.906,0.953)	0.849 (0.821,0.873)	0.786
Radiologists with SCAR-Net							
All	0.939 (0.924, 0.951)	0.941 (0.917,0.960)	0.935 (0.917,0.949)	0.871 (0.838,0.898)	0.972 (0.959,0.981)	0.937 (0.923,0.949)	0.905
Junior	0.926 (0.902, 0.945)	0.929 (0.888,0.956)	0.923 (0.896,0.944)	0.850 (0.800,0.889)	0.965 (0.945,0.979)	0.925 (0.903,0.942)	0.885
Senior	0.951 (0.934, 0.967)	0.956 (0.920,0.976)	0.946 (0.922,0.963)	0.892 (0.847,0.925)	0.979 (0.961,0.988)	0.949 (0.930,0.963)	0.923

**Table 4:** Comparison of assisted diagnostic results in the external test set

Modalities	AUC (95% CI)	TPR (95% CI)	TNR (95% CI)	PPV (95% CI)	NPV (95% CI)	ACC (95% CI)	F1
Radiologists only							
All	0.824 (0.805, 0.842)	0.775 (0.738,0.808)	0.872 (0.858,0.885)	0.589 (0.553,0.625)	0.942 (0.932,0.952)	0.854 (0.840,0.866)	0.669
Junior	0.795 (0.767, 0.822)	0.747 (0.693,0.795)	0.842 (0.820,0.862)	0.528 (0.479,0.578)	0.934 (0.917,0.947)	0.824 (0.804,0.843)	0.619
Senior	0.852 (0.827, 0.876)	0.802 (0.751,0.845)	0.902 (0.884,0.918)	0.660 (0.607,0.709)	0.951 (0.936,0.962)	0.883 (0.865,0.899)	0.724
Radiologists with Swin-Unet							
All	0.884 (0.867, 0.900)	0.833 (0.800,0.862)	0.908 (0.895,0.919)	0.681 (0.645,0.715)	0.958 (0.949,0.966)	0.894 (0.882,0.904)	0.750
Junior	0.867 (0.842, 0.890)	0.806 (0.755,0.848)	0.901 (0.883,0.917)	0.659 (0.606,0.707)	0.952 (0.937,0.963)	0.883 (0.865,0.899)	0.725
Senior	0.901 (0.881, 0.920)	0.861 (0.815,0.897)	0.914 (0.897,0.929)	0.704 (0.653,0.750)	0.965 (0.953,0.975)	0.904 (0.888,0.918)	0.774
Radiologists with SCAR-Net							
All	0.942 (0.931, 0.953)	0.934 (0.910,0.952)	0.950 (0.941,0.958)	0.816 (0.784,0.844)	0.984 (0.978,0.988)	0.947 (0.938,0.955)	0.871
Junior	0.922 (0.901, 0.941)	0.901 (0.860,0.931)	0.942 (0.927,0.954)	0.786 (0.737,0.828)	0.976 (0.965,0.983)	0.934 (0.920,0.946)	0.840
Senior	0.963 (0.950, 0.974)	0.967 (0.939,0.982)	0.958 (0.945,0.968)	0.846 (0.802,0.882)	0.992 (0.985,0.996)	0.960 (0.949,0.969)	0.903

### Segmentation performance of the model

**Figure 4:**
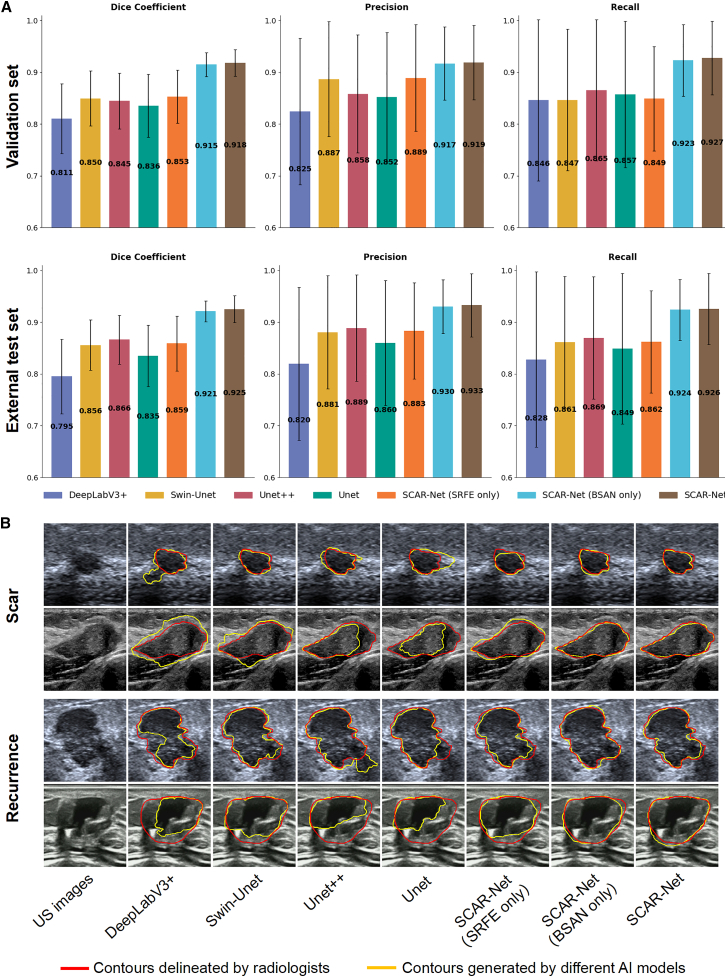
Comparison of segmentation performance and qualitative results across different models (A) Comparison of segmentation results of different models in the validation set; the first and second rows show the Dice coefficient, precision, and recall of models in the validation set and external test set, respectively. (B) Comparison of different methods for segmenting scars and recurrent lesions, where the red curves are standard curves drawn by radiologists, and yellow represents the output results of the various models.

In terms of segmentation performance, as shown in Figure 4, SCAR-Net demonstrated excellent segmentation accuracy. The quantitative analysis in Figure 4A shows that SCAR-net achieved dice coefficients of 0.918 and 0.925 in the validation and external test sets, respectively, approximately 7–8 percentage points higher than the closest Swin-Unet model (0.850/0.856), and significantly outperforming other traditional segmentation models such as DeepLabV3+ (0.795/0.811), Unet (0.836/0.835), and Unet++ (0.845/0.866).

Notably, SCAR-Net performed excellently in both the precision and recall metrics, ensuring precision while maintaining high recall rates, which is particularly important for not missing recurrent lesions in clinical practice. The visual comparison in Figure 4B more intuitively demonstrates the model’s advantages. In the scar cases in the first and second rows, the yellow segmentation contours produced by SCAR-Net (rightmost column) almost perfectly overlap with the red contours annotated by radiologists, while other models show varying degrees of over-segmentation or under-segmentation. In the recurrent lesion cases in the third and fourth rows, especially in areas with blurred boundaries and irregular morphology, SCAR-Net still precisely captured subtle changes in lesion boundaries. Additionally, ablation experiments demonstrated SCAR-Net’s performance using only SRFE or BSAN. As shown in Figure 4A, using SRFE alone showed no significant difference from baseline Swin-Unet, while using BSAN alone substantially improved segmentation performance.

### Subgroup performance analysis

We conducted comprehensive subgroup analyses that revealed distinct performance patterns across different clinical scenarios (Table 5). Notably, SCAR-Net demonstrated robust generalizability across different data balancing methods, including random down-sampling (RDS), random over-sampling (ROS), and the synthetic minority oversampling technique (SMOTE). All methods produced highly consistent results (*p* = 0.196 in the validation set and *p* = 0.233 in the external test set), with the default RDS configuration performing slightly better. SCAR-Net’s robustness also extended across ultrasound equipment manufacturers (*p* = 0.847 and *p* = 0.782, respectively), indicating that neither class imbalance mitigation strategies nor hardware variability substantially affected model performance. However, a markedly different pattern emerged when examining lesion size and image quality. While radiologists' baseline diagnostic performance showed no significant differences between lesions ≥5 mm and <5 mm (*p* = 0.209 in the validation set, pp = 0.271 in the external test set), SCAR-Net exhibited significantly superior performance for larger lesions compared to smaller ones (*p* < 0.001 in both datasets). Similarly, although radiologists showed only modest sensitivity to image quality (*p* = 0.035 and *p* = 0.016), SCAR-Net demonstrated pronounced performance degradation with blurry images (*p* < 0.001 in both datasets). Image clarity was determined by consensus among three breast radiologists based on lesion characteristics, echogenicity, and background; disagreements were resolved by voting.

**Table 5:** Subgroup analysis of the validation set and external test set

Categories	Total Number (Recurrence)	Radiologists Only AUC (95%CI)	SCAR-Net AUC (95%CI)	Radiologists with SCAR-Net AUC (95%CI)
Validation set				
Size, *p* value	–	0.209	<0.001	<0.001
≥5 mm	144 (48)	0.818 (0.793, 0.841)	0.976 (0.958, 0.989)	0.952 (0.928, 0.970)
<5 mm	92 (27)	0.803 (0.771, 0.833)	0.943 (0.913, 0.966)	0.915 (0.881, 0.942)
Machine, *p* value	–	0.612	0.847	0.791
Toshiba	75 (24)	0.815 (0.786, 0.848)	0.969 (0.943, 0.986)	0.941 (0.910, 0.964)
GE	71 (23)	0.805 (0.771, 0.836)	0.965 (0.937, 0.983)	0.937 (0.905, 0.961)
Siemens	90 (28)	0.807 (0.778, 0.834)	0.966 (0.941, 0.983)	0.933 (0.907, 0.958)
Balancing Methods, *p* value	–	–	0.196	–
RDS	236 (75)	/	0.967 (0.942, 0.988)	/
ROS	236 (75)	/	0.936 (0.906, 0.959)	/
SMOTE	236 (75)	/	0.941 (0.912, 0.963)	/
Image clarity, *p* value	–	0.035	<0.001	<0.001
Clear	179 (57)	0.823 (0.799, 0.846)	0.978 (0.962, 0.989)	0.954 (0.936, 0.967)
Blurry	57 (18)	0.779 (0.739, 0.816)	0.932 (0.893, 0.961)	0.901 (0.859, 0.934)
External test set				
Size, *p* value	–	0.271	<0.001	<0.001
≥5 mm	307 (59)	0.831 (0.810, 0.851)	0.975 (0.969, 0.980)	0.968 (0.958, 0.976)
<5 mm	169 (32)	0.813 (0.786, 0.838)	0.946 (0.933, 0.957)	0.912 (0.898, 0.930)
Machine, *p* value	–	0.568	0.782	0.794
Toshiba	165 (32)	0.829 (0.807, 0.854)	0.968 (0.960, 0.975)	0.945 (0.932, 0.956)
GE	148 (28)	0.819 (0.793, 0.843)	0.963 (0.954, 0.971)	0.950 (0.936, 0.962)
Siemens	163 (31)	0.822 (0.797, 0.846)	0.966 (0.959, 0.973)	0.948 (0.928, 0.953)
Balancing Methods, *p* value	–	–	0.233	–
RDS	476 (91)	/	0.965 (0.958, 0.967)	/
ROS	476 (91)	/	0.943 (0.935, 0.950)	/
SMOTE	476 (91)	/	0.947 (0.939, 0.956)	/
Image clarity, *p* value	–	0.016	<0.001	<0.001
Clear	404 (77)	0.833 (0.816, 0.855)	0.977 (0.972, 0.982)	0.959 (0.951, 0.967)
Blurry	72 (14)	0.796 (0.758, 0.825)	0.925 (0.903, 0.943)	0.898 (0.871, 0.921)

The clinical implications of these findings become evident when examining AI-assisted diagnostic performance. When radiologists incorporated SCAR-Net’s recommendations, their diagnostic accuracy reflected the model’s sensitivity patterns rather than their own baseline characteristics. Specifically, AI-assisted AUCs showed significant differences based on lesion size and image quality (all *p* < 0.001), mirroring SCAR-Net’s performance trends rather than radiologists’ original diagnostic patterns. This observation suggests that radiologists’ decision-making was substantially influenced by the model’s confidence levels, which varied with lesion size and image clarity. These findings underscore an important consideration for clinical implementation: While SCAR-Net provides substantial diagnostic improvements across diverse technical platforms and balancing strategies, its effectiveness depends critically on obtaining high-quality images and may be limited for very small lesions (<5 mm), where additional imaging modalities or closer clinical follow-up may be warranted.

## Discussion

This study developed SCAR-Net, the first DL model specifically designed to differentiate between postoperative scars and recurrent lesions in breast cancer. Our results demonstrate that this model not only performed excellently in lesion segmentation tasks but also effectively improved radiologists’ diagnostic accuracy and efficiency, especially for radiologists with less experience.

While DL models for breast ultrasound have shown significant progress in recent years, the specific challenge of differentiating postoperative scars from recurrent lesions remains largely unaddressed in the existing literature. To our knowledge, only one single-center study has reported the use of machine learning-based ultrasound radiomics models for predicting breast cancer recurrence risk, achieving AUCs of 0.817–0.851.^33^ However, this study focused on predicting future recurrence risk rather than identifying existing recurrent lesions versus scars. For breast tumor segmentation in general, recent studies have reported Dice similarity coefficients ranging from 0.79 to 0.86.^34^ In contrast to conventional DL architectures previously applied to breast ultrasound segmentation,^35^^,^^36^^,^^37^^,^^38^^,^^39^ SCAR-Net is designed to enhance boundary delineation and texture representation, thereby achieving more accurate differentiation between postoperative scars and recurrent lesions. It consequently demonstrates competitive segmentation performance, with Dice coefficients of 0.918–0.925, despite the inherent complexity of distinguishing postoperative scars, which has seldom been addressed in prior segmentation research. Beyond segmentation, SCAR-Net maintained robust diagnostic capability, achieving AUCs of 0.965–0.967 for differentiating postoperative scars from recurrent lesions.

To evaluate SCAR-Net’s clinical interpretability, all participating radiologists scored the AI-generated segmentation and feature heatmaps on a 0–5 scale (0 being the worst, 5 being the best). Analysis of cases where radiologists altered their diagnoses after AI assistance revealed that segmentation quality was significantly higher in correctly altered versus wrongly altered cases (3.82 vs. 2.39 in validation set, 3.78 vs. 2.51 in external test set; both *p* < 0.001), while heatmap scores remained comparable between groups (*p* > 0.3), detailed information can be found in Table S9. These findings indicate that accurate lesion boundary delineation serves as a critical interpretable cue guiding appropriate diagnostic revisions. Importantly, the results also suggest that when segmentation quality is poor, the accompanying feature heatmaps may be unreliable and potentially misleading to radiologists, even though they appeared visually acceptable. This underscores the importance of precise segmentation not only as a technical metric but as a key indicator of overall model reliability in AI-assisted decision-making for distinguishing postoperative scars from recurrent lesions.

Our study demonstrates the significant value of AI technology in enhancing radiologists’ diagnostic capabilities. Detailed multi-reader, multi-case (MRMC)^40^ results are summarized in Tables S6–S8. The comprehensive reading results from six radiologists with different experience levels demonstrate that the model can effectively improve diagnostic performance across all experience levels. For junior radiologists (*n* = 3), the mean AUC increased from 0.794 (95% CI: 0.765, 0.820) under independent reading to 0.928 (95% CI: 0.905, 0.948) with AI assistance, representing a substantial improvement of 0.134 (*p* < 0.001). For senior radiologists (*n* = 3), the mean AUC improved from 0.838 (95% CI: 0.812, 0.861) to 0.954 (95% CI: 0.937, 0.968), with an increase of 0.116 (*p* < 0.001). Notably, junior radiologists showed greater overall improvement (ΔAUC = 0.134 vs. 0.116), as they are sometimes less confident in their diagnoses and may benefit more from AI guidance. Both junior and senior radiologists benefited consistently from SCAR-Net assistance, with no significant reader AI interaction effect (*p* = 0.41), suggesting that the improvement was stable across different experience levels.

We identified that radiologists’ prior knowledge might create an anchoring bias when using AI assistance. To quantify these potential biases, we conducted additional analyses examining cases where AI and radiologists initially disagreed. In the validation set, junior radiologists changed their opinions in 88.1% (31.7/36) of disagreement cases, while senior radiologists did so in 77.4% (28.7/37.1) of cases. Similar patterns were observed in the external test set, where junior radiologists changed their opinions in 83.4% (55.4/66.4) of disagreement cases, while senior radiologists changed their opinions in 74.2% (39.3/53) of cases. These findings suggest that junior radiologists demonstrated higher overall acceptance of AI recommendations compared to senior radiologists. The higher acceptance rate among junior radiologists may reflect both greater trust in AI assistance and recognition of knowledge gaps, while senior radiologists’ more selective acceptance pattern suggests greater reliance on their clinical experience when evaluating AI suggestions.

The SCAR-Net model developed in this study demonstrates the potential of DL technology in addressing the clinical challenge of differentiating between postoperative scars and recurrent lesions in breast cancer. It can be integrated into existing clinical workflows through several approaches. For instance, it can be directly embedded into PACS as a parallel reading tool that automatically analyzes postoperative breast ultrasound examinations, rapidly pre-populating assessment categories and automatically flagging high-risk targets and regions forradiologists’ priority review. Alternatively, it can serve as an on-demand second opinion tool, providing probability scores and lesion-level visual explanations to support radiologists’ in re-evaluating ambiguous findings and prioritizing suspicious regions. Future research should explore these different implementation strategies through clinical workflow studies to determine which integration approach maximizes benefits while minimizing disruption to existing practice. Despite its strong performance, certain imaging scenarios remain challenging for SCAR-Net. Extremely small recurrent lesions, heterogeneous postoperative architectural distortion, extensive fibrotic scar tissue, or ultrasound images with low signal-to-noise ratio and strong acoustic shadowing may reduce prediction reliability and increase diagnostic uncertainty. SCAR-Net is therefore intended as a decision-support tool for evaluating suspicious lesions in the postoperative surgical bed during routine ultrasound follow-up, and its outputs should be interpreted in conjunction with clinical context and radiologist expertise.

Future research directions should include the multimodal integration of SCAR-Net with other imaging modalities. While ultrasound offers excellent accessibility and cost-effectiveness for routine follow-up, combining information from ultrasound, MRI, and mammography holds promise for further improving the accuracy and robustness of recurrence diagnosis. MRI provides superior soft tissue contrast and sensitivity for detecting early recurrent lesions, while mammography offers complementary information through its ability to identify suspicious calcifications and architectural distortions. Moreover, developing multimodal AI models capable of comprehensively processing medical imaging information across different time points and imaging modalities would better align with the comprehensive assessment approach employed in clinical practice, where radiologists routinely integrate longitudinal findings from multiple modalities to inform diagnostic decisions.

### Limitations of the study

This study has several limitations that warrant consideration. First, the absence of longitudinal validation represents a significant gap, as post-surgical tissue changes evolve considerably over time and the characteristics of both scar tissue and recurrent lesions may shift substantially during follow-up. Although we attempted to collect serial imaging data from the same patients, insufficient longitudinal datasets were obtained due to inconsistent follow-up schedules and fragmented imaging records across institutions, preventing the evaluation of SCAR-Net’s temporal robustness. We have initiated a prospective longitudinal study to address this limitation. Second, all participating centers were located in China, which may limit generalizability to populations with different demographic and genetic characteristics. Third, the retrospective design limits our ability to assess real-world implementation, and prospective validation in routine clinical workflows is needed. Fourth, as ultrasound is operator-dependent, differences in probe handling and acquisition settings may affect image consistency. Although our multicenter dataset, collected by multiple radiologists, helps mitigate this bias, residual inter-operator variability cannot be fully eliminated.

Finally, SCAR-Net currently operates exclusively on ultrasound images, while clinical practice often integrates multiple imaging modalities. Future research should explore multimodal integration of ultrasound with MRI and mammography to enhance diagnostic accuracy and better align with comprehensive clinical assessment practices. Additionally, expanding the dataset to include geographically diverse populations and continuously incorporating additional recurrence cases would strengthen the model’s generalizability and performance on challenging scenarios. These efforts, combined with prospective clinical workflow studies, will be essential for validating SCAR-Net’s real-world utility and guiding its safe implementation in postoperative breast cancer surveillance.

## Resource availability

### Lead contact

Further information and requests for resources should be directed to and will be fulfilled by the lead contact, Yong Wu (wuyong@zjcc.org.cn).

### Materials availability

This study did not generate new unique reagents.

### Data and code availability

• The ultrasound data reported in this paper will be shared by the lead contact upon request.
• All original code has been deposited at GitHub (https://github.com/seista131/SCAR-Net) and is publicly available as of the date of publication. DOI is listed in the key resources table.
• Any additional information required to reanalyze the data reported in this paper is available from the lead contact upon request.

## Acknowledgments

The authors would like to thank the radiologist teams of the participating hospitals for their hard work. This work was supported by the Research Program of Zhejiang Provincial Department of Health (2022KY699, 2023KY561, 2025KY1676), the Pioneer and Leading Goose R&D Program of Zhejiang (2023C04039), and the Research Program of Zhejiang Cancer Hospital (PY2023018).

## Author contributions

Conceptualization: Y.W.; methodology: J.Y.; writing – original draft: N.F.; Writing-Review and Editing: N.F., C.Y., D.X.; visualization: N.F., S.Z., Z.L., and D.Y.; investigation: N.F., S.Z., Z.L., D.Y., X.Z., X.L., W.Z., J.Z., and L.C.; data curation: N.F., S.Z., Z.L., X.Z., X.L., W.Z., J.Z., and L.C.; validation: J.Y.; supervision: C.Y., D.X., and Y.W.; project administration: C.Y., D.X., Y.W.; funding acquisition: N.F., D.Y., C.Y., and D.X. All authors reviewed the whole work and approved the final version of the manuscript.

## Declaration of interests

The authors declare no competing interests.

## STAR★Methods

### Key resources table

**Table:** 

REAGENT or RESOURCE	SOURCE	IDENTIFIER
**Software and algorithms**		

Python 3.9	Python	https://www.python.org
PyTorch 2.5.1 PyTorch	PyTorch	https://pytorch.org
SPSS 25.0 SPSS	SPSS	https://spss.en.softonic.com/

**Deposited data**		

SCAR-Net code	This paper	https://github.com/seista131/SCAR-Net

### Experimental model and study participant details

#### AI models

The SCAR-Net model proposed in this study adopts Swin-Unet as the backbone network. Considering that accurate scar identification faces a dual challenge: it requires not only precise spatial localization of scar boundaries, but also robust feature extraction to distinguish between scars and recurrent lesions with overlapping characteristics. To address this dual challenge, we designed a framework integrating two dedicated modules: the Boundary-Sensitive Attention Network (BSAN) and the Scar-Recurrence Feature Enhancer (SRFE). Specifically, the BSAN module aims to enhance segmentation accuracy by incorporating boundary-aware attention mechanisms into cross-layer connections, thereby better segmenting fine structural details during feature propagation. The SRFE module focuses on improving classification accuracy through a more powerful feature extraction module.

More specifically, the BSAN module is inserted into the skip connections between the Transformer encoder and decoder. This module was designed because boundary features are the most critical yet most challenging features for distinguishing between these two lesions. Sometimes scars may also exhibit irregular borders characteristic of malignant lesions, while early recurrent lesions may present seemingly benign clear boundaries. Traditional segmentation networks treat boundaries as a byproduct of region segmentation rather than independent diagnostic features. Therefore, the presence of BSAN can focus on regions with significant gradient changes, guiding the model to attend to the boundary regions of scars and recurrent lesions, thereby effectively enhancing segmentation accuracy and preserving fine structural details during feature propagation. The SRFE module is introduced in the final stage of the decoder, aiming to address the significant overlap in internal texture patterns. Since both scars and recurrent lesions may exhibit heterogeneous echoes and posterior acoustic shadowing, the presence of this module strengthens the backend feature recognition capability of the model, thereby better improving classification accuracy.

Finally, a Multi-Feature Transformer Layer (MFTL) is used to aggregate features and complete the diagnostic task. Detailed designs of SCAR-Net and its BSAN, SRFE, and MFTL subnetworks are referenced in Tables S2–S5. Additionally, a comprehensive network architecture diagram is provided in Figure S3 for visual illustration.

#### Participant details

This multicenter retrospective study received ethical approval from institutional review boards (IRBs) at all participating centers. The study strictly followed the ethical principles of the Declaration of Helsinki regarding research involving human participants, implemented rigorous measures to ensure patient privacy and data security, and anonymized all patient data including medical images before analysis. Due to the retrospective nature of the study, the ethics committees waived the requirement for informed consent from patients. The hospitals participating in this multicenter study and their ethics committee approval numbers are as follows: Zhejiang Cancer Hospital (IRB-2022-738), Zhejiang Provincial Hospital of Chinese Medicine (IRB-KL-063-01), The First People's Hospital of Lin'an (IRB-2022-72) and Shaoxing People's Hospital (IRB-026-Y-01).

### Method details

#### Study design

In this multicenter retrospective study, we collected a total of 34,376 postoperative follow-up ultrasound images from 5,710 breast cancer patients across four medical centers. All included patients had previously undergone surgical treatment for breast cancer and subsequently received routine ultrasound follow-up examinations. Each ultrasound image was reviewed and annotated by experienced radiologists, who identified and labeled the region of interest (ROI) as either postoperative scar tissue or recurrent lesion based on pathological confirmation and/or multidisciplinary clinical consensus.

Prior to model training, all ROI images were automatically extracted and standardized in size. Each image was encoded using one-hot labels according to the ground truth (scar or recurrence). Data augmentation techniques were employed to expand the training set and increase model robustness, including random scaling (0.9 to 1.1 times), random brightness adjustment (0.9 to 1.1 times), random rotation (-10°to +10°), horizontal and vertical flipping, addition of Gaussian noise, and random cropping.

The proposed SCAR-Net model architecture incorporated two modules: the Scar-Recurrence Feature Enhancer (SRFE) to improve texture feature extraction for both scar tissue and recurrent lesions, and the Boundary-Sensitive Attention Network (BSAN) to enhance segmentation accuracy. To address the class imbalance issue in DL datasets, we implemented a multi-round random down-sampling strategy for the majority class. This approach systematically reduced the over-representation of dominant categories while preserving their distributional characteristics; the specific sampling process is detailed in Figure S2. To improve model performance, we employed a combined loss function of Dice loss and focal Tversky loss to enhance learning of boundary regions. The model was trained using the Adam optimizer with an initial learning rate of 0.0001, along with L2 regularization with a weight decay of 0.0001 and an early stopping strategy to prevent overfitting.

#### Data acquisition and preprocessing

Ultrasound images were acquired using multiple equipment brands including Toshiba Aplio 500 (Toshiba Medical Systems, Tokyo, Japan), GE LOGIQ E9 (GE Healthcare, Chicago, IL, USA), and Siemens ACUSON Sequoia (Siemens Healthineers, Erlangen, Germany). To account for potential variations in image quality across different devices, we implemented a comprehensive preprocessing pipeline: All images were resampled to a standard resolution of 0.1 mm/pixel to compensate for different spatial resolutions across equipment. Z-score normalization was applied to standardize the grayscale intensity distribution, reducing the impact of different gain settings and dynamic ranges among devices. All collected medical images underwent anonymization processing.

#### Image labeling and reading

All ultrasound image delineation and annotation tasks were completed by three senior radiologists using the open-source software LabelMe. The radiologists participating in the image delineation task all had over 8 years of experience in breast ultrasound diagnosis. After one radiologist completed the delineation, another radiologist was required to review the delineation results. If discrepancies arose, a third radiologist would intervene and arbitrate the delineation results to ensure that all regions of interest (ROI) contour annotations were accurate and reliable. The ROIs were normalized to a standard size of 224×224 pixels using an augmented annotation method to ensure that the ROIs maintained their original proportions; details of the augmentation method are shown in Figure S1. Clinical data for each case were also systematically collected, including patient age, lesion size, and other relevant clinical information.

After model training was completed, six radiologists independently reviewed the images using a cross-randomized reading order. Each radiologist first conducted an independent diagnosis phase, then entered the AI-assisted diagnosis phase, with a four-week wash-out period before assisted diagnosis to avoid memory bias. Each radiologist read the same case set in both phases (with different randomized orders) and remained blinded to case numbers and pathological results. For statistical analysis, we followed the MRMC framework for analysis, calculating the mean AUC and 95% confidence intervals under multi-reader, multi-case conditions, and comparing the diagnostic performance differences between independent and AI-assisted reading conditions, thereby controlling for reader-case related confounding factors. During AI-assisted reading, the radiologist's workstation simultaneously displayed the original ultrasound image, contour curves indicating the AI-segmented lesion region of interest, the quantified recurrence risk probability value output by the model, and feature heatmaps.

### Quantification and statistical analysis

Area under the receiver operating characteristic curve (AUC), sensitivity (Sen), specificity (Spec), accuracy (Acc), positive predictive value (PPV), negative predictive value (NPV), and F1 score were used to evaluate the performance of the SCAR-Net model. For the segmentation task, the Dice similarity coefficient, precision, and recall were used as the primary evaluation metrics. In the clinical evaluation, six radiologists with different experience levels (three junior and three senior) participated in a two-stage diagnostic experiment. In the first stage, the six radiologists performed independent diagnoses; in the second stage, all radiologists diagnosed with AI assistance (including observation of AI output risk probability, segmentation results, and feature heat maps).
