# Supplementary material for: SCAR-Net-assisted ultrasound diagnosis of postoperative scars and recurrent lesions in breast cancer
Source: iScience. 2026 Apr 1;29(5):115550. doi: 10.1016/j.isci.2026.115550 (PMC13098497; doi:10.1016/j.isci.2026.115550)
Supplement: Document S1. Figures S1–S4 and Tables S1–S11 [file mmc1.pdf]

iScience, Volume *29*

Supplemental information

SCAR-Net-assisted ultrasound diagnosis of postoperative scars and recurrent lesions in breast cancer

Na Feng, Shanshan Zhao, Zhikai Lei, Dan Yi, Jincao Yao, Xiao Zhang, Xiangyang Li, Weijie Zou, Jian Zhang, Liyu Chen, Chen Yang, Dong Xu, and Yong Wu

SUPPLEMENTAL INFORMATION

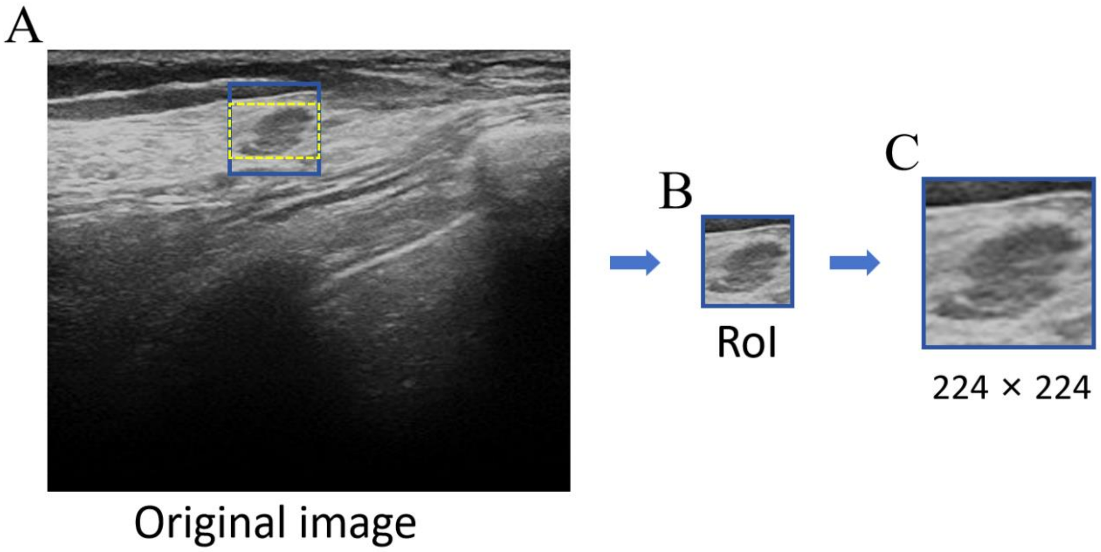

Figure S1. Flowchart of the lesion's image normalization process. Related to Figure 1. For each ultrasound image, we first annotated the bounding box (BBox) of the lesion, then employed a proportional method that preserved the lesion's aspect ratio during normalization. The process includes: (A) Annotated bounding of the lesion by a yellow rectangle whose shorter edges were extended to produce the blue square; (B) Extracted square-shaped region of interest (RoI); (c) Normalized square image to 224×224.

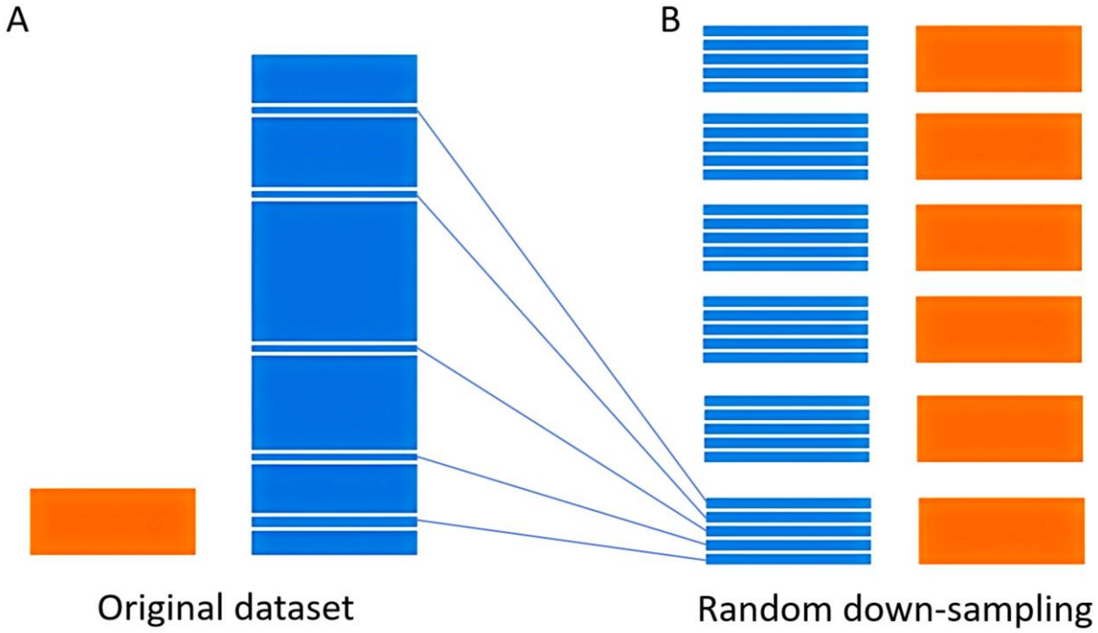

Figure S2. Schematic illustration of random down-sampling strategy for addressing class imbalance in deep learning. Related to Figure 2. (A) The original dataset shows class imbalance with the majority class (blue) significantly outnumbering the minority class (orange). (B) Random down-sampling is applied to reduce the majority class samples while preserving all minority class samples, creating a more balanced dataset for model training. This approach helps prevent bias toward the majority class and improves performance on underrepresented classes.

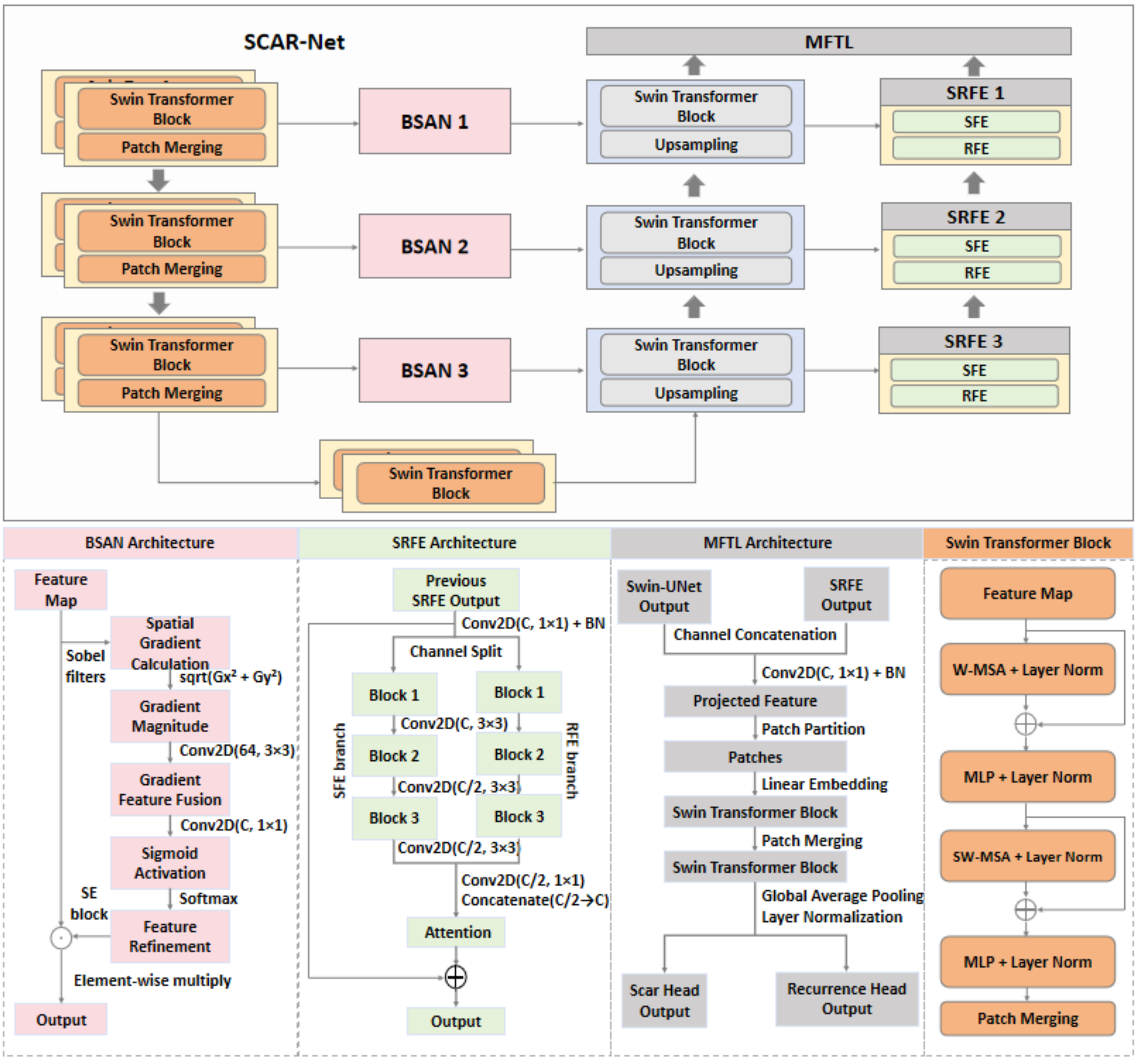

Figure S3. Overall architecture of SCAR-Net. Related to Figure 2. The network consists of four main modules: SCAR-Net backbone with Swin Transformer Blocks for hierarchical feature extraction, BSAN (Boundary-Sensitive Attention Network) for feature enhancement, MFTL (Multi-Feature Transformer Layer) for multi-scale feature fusion, and SRFE (Scar-Recurrence Feature Enhancer) for high-quality output generation. Detailed designs of SCAR-Net and its BSAN, SRFE, and MFTL subnetworks are referenced in Supplementary Tables S2 to S5.

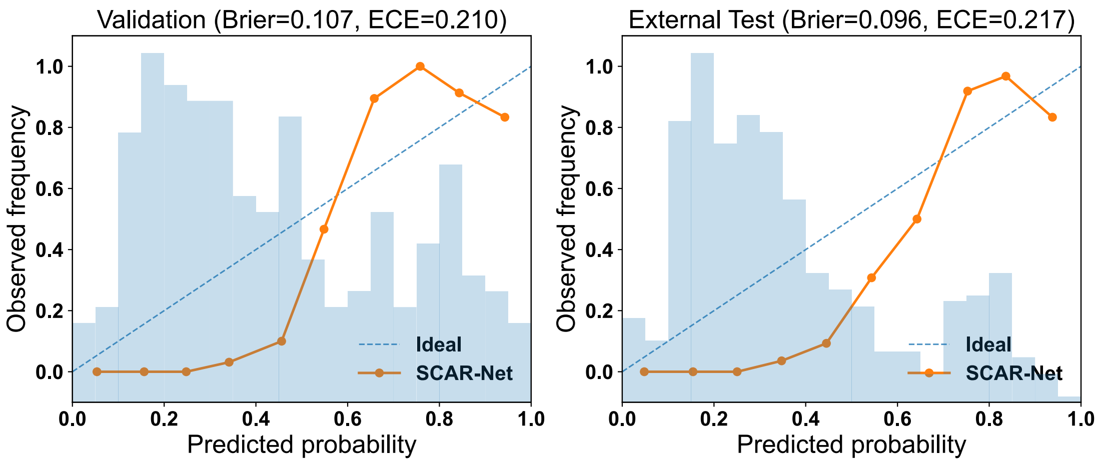

Figure S4. Calibration of SCAR-Net probability outputs. Related to Figure 3. Left panel = validation set; right panel = external test set. Orange line with dots = reliability curve; blue dashed line = ideal calibration. Light-blue histogram = distribution of predicted probabilities. Reported Brier score and expected calibration error (ECE) are shown in the panel titles.

Table S1. Multi-center data information. Related to Table 1.

Category	Center 1	Center 2	Center 3	Center 4
No. of the images	29542	2876	1068	890
No. of the lesions	5146	543	261	215
No. of the patients	4757	510	240	203
Age, mean (SD)	47(8)	49 (9)	46 (10)	51 (9)
Size, mm (SD)	9 (4)	9 (5)	8 (4)	9 (5)
Scar	4599	472	212	173
Recurrence	547	71	49	42

SD: standard deviation; Center 1: Zhejiang Cancer Hospital; Centre 2: Shaoxing People's Hospital; Center 3: Zhejiang Provincial Hospital of Chinese Medicine; Center 4: the First People's Hospital of Lin'an.

Table S2. Overall SCAR-Net architecture. Related to Figure 2.

Layer/Module	Output Size	Operations
Input Image	224×224×3	-
Patch Partition	56×56×96	Patch size 4×4, embedding dim 96
Encoder Stage 1	56×56×96	Swin Transformer Block (window=7, heads=3)<br>Patch Merging
BSAN 1	56×56×96	Boundary-sensitive attention operation
Encoder Stage 2	28×28×192	Swin Transformer Block (window=7, heads=6)<br>Patch Merging
BSAN 2	28×28×192	Boundary-sensitive attention operation
Encoder Stage 3	14×14×384	Swin Transformer Block (window=7, heads=12)<br>Patch Merging
BSAN 3	14×14×384	Boundary-sensitive attention operation
Bottleneck	7×7×768	Swin Transformer Block (window=7, heads=24)
Decoder Stage 3	14×14×384	Swin Transformer Block (window=7, heads=12)<br>Upsampling
SRFE 3	14×14×384	SFE + RFE modules
Decoder Stage 2	28×28×192	Swin Transformer Block (window=7, heads=6)<br>Upsampling
SRFE 2	28×28×192	SFE + RFE modules
Decoder Stage 1	56×56×96	Swin Transformer Block (window=7, heads=3)<br>Upsampling
SRFE 1	56×56×96	SFE + RFE modules
Projection	56×56×96	Multi-feature fusion transformer
Upsampling	224×224×1	Linear projection + Upsampling
MTL	Cls	Cls = num_classes

Table S3. Boundary sensitive attention network (BSAN) architecture. Related to Figure 2.

**Layer**	**Output Size**	**Description**
Input Feature Map	H×W×C	Feature map from encoder, C = channals
Spatial Gradient Calculation	H×W×2	Sobel operators for x and y directions (3×3 kernels)
Gradient Magnitude	H×W×1	Sqrt(Gx^2 + Gy^2)
Gradient Feature Extraction	H×W×64	Conv2D(1→64, kernel=3×3) + BatchNorm + ReLU
Gradient-Feature Fusion	H×W×C	Conv2D(64→C, kernel=1×1) + BatchNorm
Sigmoid Activation	H×W×C	Soft attention map
Feature Refinement	H×W×C	Channel attention with SE block
Attention Application	H×W×C	Element-wise multiplication with input feature
Residual Connection	H×W×C	Add original input features

Table S4. Scar-Recurrence Feature Enhancer (SRFE) Architecture. Related to Figure 2.

Component	Layer	Output Size	Description
Decoder Input	Swin-UNet Decoder Output	H×W×C	Features from corresponding Swin-UNet decoder stage
Previous SRFE Input	Upsampled Previous SRFE	H×W×C	Previous SRFE output upsampled to current resolution (except for SRFE 3)
Feature Fusion	Concatenation & Projection	H×W×C	Concatenate both inputs and project back to C channels (Conv2D + BatchNorm)
Channel Split	Feature Distribution	H×W×C	Distribute features to SFE and RFE paths
Scar Feature Enhancer (SFE)	Block 1	H×W×C/2	Conv2D(C→C/2, kernel=3×3) + BatchNorm + ReLU
	Block 2	H×W×C/2	Conv2D(C/2→C/2, kernel=3×3) + BatchNorm + ReLU
	Block 3	H×W×C/2	Conv2D(C/2→C/2, kernel=3×3) + BatchNorm + ReLU
	Output Projection	H×W×C/2	Conv2D(C/2→C/2, kernel=1×1) + BatchNorm
Recurrence Feature Enhancer (RFE)	Block 1	H×W×C/2	Conv2D(C→C/2, kernel=3×3) + BatchNorm + ReLU
	Block 2	H×W×C/2	Conv2D(C/2→C/2, kernel=3×3) + BatchNorm + ReLU
	Block 3	H×W×C/2	Conv2D(C/2→C/2, kernel=3×3) + BatchNorm + ReLU
	Output Projection	H×W×C/2	Conv2D(C/2→C/2, kernel=1×1) + BatchNorm
Feature Integration	Branch Concatenation	H×W×C	Concatenate SFE and RFE outputs along channel dimension
	Feature Fusion	H×W×C	Channel-wise attention (SE block) to balance feature importance
	Residual Connection	H×W×C	Add with original fused input features

Table S5. Multi-feature Transformer Layer (MFTL) Architecture. Related to Figure 2.

Component	Layer	Output Size	Description
Backbone Output	Feature Map	$224 \times 224 \times C_1$	Final output feature map from Swin-UNet (after upsampling)
SRFE Output	Feature Map	$224 \times 224 \times C_2$	Final output from SRFE module chain (upsampled to original resolution)
Channel Concatenation	Concatenation	$224 \times 224 \times (C_1 + C_2)$	Concatenating Swin-UNet and SRFE features along channel dimension
Feature Projection	Conv2D	$224 \times 224 \times C_3$	1×1 convolution to project concatenated features to appropriate channel dimension C_3
Patch Partitioning	Patch Partition	$56 \times 56 \times 16C_3$	Dividing feature map into 4×4 patches
Linear Embedding	Linear Projection	56×56×D	Linear layer mapping each patch to D-dimensional embedding space
Swin Transformer Block 1	2×Swin Block	56×56×D	Swin Transformer blocks with W-MSA and SW-MSA, maintaining resolution
Patch Merging	Downsampling	28×28×2D	Merging adjacent patches, reducing resolution and increasing channels
Swin Transformer Block 2	2×Swin Block	28×28×2D	Second stage Swin Transformer blocks with larger receptive field
Global Pooling	Global Avg Pool	1×1×2D	Global average pooling to compress features into a single vector
Feature Normalization	LayerNorm	1×1×2D	Layer normalization of global feature vector
Scar Binary Output	Dense + Sigmoid	1×1×1	Fully connected layer with sigmoid activation (1=scar present, 0=no scar)
Recurrence Binary Output	Dense + Sigmoid	1×1×1	Fully connected layer with sigmoid activation (1=recurrence present, 0=no recurrence)

Table S6. Diagnostic performance of each radiologist in the validation set. J1 and S1 represent junior radiologist 1 and senior radiologist 1 respectively. Related to Figure 4.

Doctor	Method	TPR	TNR	PPV	NPV	ACC	F1
J1	Independent	0.707<br>（0.608，0.809）	0.870<br>（0.814，0.919）	0.716<br>（0.609，0.810）	0.864<br>（0.810，0.918）	0.815<br>（0.769，0.863）	0.711
	with Swin-Unet	0.787<br>（0.694，0.875）	0.832<br>（0.773，0.888）	0.686<br>（0.588，0.782）	0.893<br>（0.841，0.939）	0.818<br>（0.771，0.865）	0.733
	with SCAR-Net	0.907<br>（0.833，0.969）	0.919<br>（0.875，0.958）	0.840<br>（0.754，0.917）	0.955<br>（0.920，0.986）	0.915<br>（0.877，0.949）	0.872
J2	Independent	0.733<br>（0.629，0.825）	0.770<br>（0.704，0.832）	0.598<br>（0.500，0.705）	0.861<br>（0.800，0.913）	0.758<br>（0.703，0.814）	0.659
	with Swin-Unet	0.813<br>（0.720，0.903）	0.807<br>（0.741，0.871）	0.663<br>（0.570，0.765）	0.903<br>（0.850，0.949）	0.809<br>（0.758，0.860）	0.731
	with SCAR-Net	0.947<br>（0.890，0.989）	0.932<br>（0.887，0.967）	0.866<br>（0.786，0.935）	0.974<br>（0.947，0.994）	0.936<br>（0.903，0.966）	0.904
J3	Independent	0.800<br>（0.705，0.889）	0.857<br>（0.803，0.908）	0.723<br>（0.622，0.816）	0.902<br>（0.853，0.947）	0.839<br>（0.792，0.886）	0.759
	with Swin-Unet	0.853<br>（0.773，0.929）	0.832<br>（0.775，0.891）	0.703<br>（0.610，0.798）	0.924<br>（0.881，0.964）	0.839<br>（0.797，0.886）	0.771
	with SCAR-Net	0.933<br>（0.870，0.985）	0.919<br>（0.877，0.957）	0.843<br>（0.771，0.917）	0.967<br>（0.936，0.993）	0.924<br>（0.890，0.958）	0.886
S1	Independent	0.773<br>（0.676，0.864）	0.882<br>（0.828，0.928）	0.753<br>（0.653，0.841）	0.893<br>（0.845，0.935）	0.847<br>（0.801，0.890）	0.763
	with Swin-Unet	0.880<br>（0.800，0.948）	0.857<br>（0.801，0.910）	0.742<br>（0.651，0.831）	0.939<br>（0.900，0.975）	0.864<br>（0.822，0.907）	0.805
	with SCAR-Net	0.947<br>（0.887，0.989）	0.944<br>（0.908，0.976）	0.887<br>（0.818，0.953）	0.974<br>（0.946，0.994）	0.945<br>（0.915，0.970）	0.916
S2	Independent	0.827<br>（0.739，0.907）	0.795<br>（0.732，0.857）	0.653<br>（0.560，0.745）	0.908<br>（0.857，0.950）	0.805<br>（0.754，0.852）	0.729
	with Swin-Unet	0.853<br>（0.770，0.927）	0.839<br>（0.782，0.894）	0.711<br>（0.615，0.806）	0.925<br>（0.879，0.964）	0.843<br>（0.797，0.890）	0.776
	with SCAR-Net	0.960<br>（0.909，0.991）	0.957<br>（0.921，0.987）	0.911<br>（0.841，0.971）	0.981<br>（0.956，0.996）	0.958<br>（0.932，0.983）	0.935
S3	Independent	0.853<br>（0.773，0.924）	0.857<br>（0.796，0.907）	0.736<br>（0.639，0.822）	0.926<br>（0.880，0.962）	0.856<br>（0.805，0.898）	0.790
	with Swin-Unet	0.880<br>（0.793，0.950）	0.820<br>（0.751，0.878）	0.695<br>（0.600，0.790）	0.936<br>（0.887，0.973）	0.839<br>（0.788，0.886）	0.776
	with SCAR-Net	0.963<br>（0.911，0.993）	0.938<br>（0.901，0.974）	0.878<br>（0.807，0.948）	0.981<br>（0.955，0.997）	0.945<br>（0.915，0.970）	0.917

Table S7. Diagnostic performance of each radiologist in the external test set. J1 and S1 represent junior radiologist 1 and senior radiologist 1 respectively. Related to Figure 4.

Doctor	Method	TPR	TNR	PPV	NPV	ACC	F1
J1	Independent	0.714（0.619，0.809）	0.860（0.821，0.892）	0.546（0.453，0.636）	0.927（0.901，0.955）	0.832（0.794，0.868）	0.619
	with Swin-Unet	0.780（0.691，0.856）	0.891（0.857，0.922）	0.628（0.544，0.713）	0.945（0.920，0.965）	0.870（0.836，0.899）	0.696
	with SCAR-Net	0.890（0.823，0.949）	0.938（0.912，0.960）	0.771（0.693，0.848）	0.973（0.955，0.989）	0.929（0.903，0.950）	0.827
J2	Independent	0.747（0.656，0.833）	0.816（0.774，0.851）	0.489（0.404，0.569）	0.932（0.904，0.957）	0.803（0.767，0.836）	0.591
	with Swin-Unet	0.802（0.710，0.879）	0.901（0.870，0.930）	0.658（0.564，0.741）	0.951（0.925，0.972）	0.882（0.851，0.910）	0.723
	with SCAR-Net	0.901（0.837，0.956）	0.945（0.923，0.965）	0.796（0.716，0.865）	0.976（0.960，0.989）	0.937（0.916，0.958）	0.845
J3	Independent	0.780（0.697，0.863）	0.852（0.816，0.891）	0.555（0.475，0.643）	0.943（0.919，0.965）	0.838（0.807，0.872）	0.648
	with Swin-Unet	0.835（0.757，0.906）	0.912（0.883，0.939）	0.691（0.602，0.776）	0.959（0.937，0.978）	0.897（0.868，0.922）	0.756
	with SCAR-Net	0.912（0.845，0.967）	0.943（0.918，0.966）	0.790（0.706，0.868）	0.978（0.962，0.992）	0.937（0.914，0.960）	0.847
S1	Independent	0.802（0.721，0.876）	0.919（0.892，0.945）	0.702（0.611，0.787）	0.952（0.929，0.971）	0.897（0.870，0.922）	0.749
	with Swin-Unet	0.857（0.773，0.926）	0.922（0.895，0.949）	0.722（0.642，0.804）	0.965（0.943，0.981）	0.910（0.882，0.935）	0.784
	with SCAR-Net	0.956（0.907，0.991）	0.938（0.912，0.959）	0.784（0.705，0.854）	0.989（0.978，0.997）	0.941（0.918，0.960）	0.861
S2	Independent	0.813（0.729，0.889）	0.878（0.844，0.910）	0.612（0.525，0.703）	0.952（0.928，0.974）	0.866（0.834，0.895）	0.698
	with Swin-Unet	0.846（0.767，0.917）	0.914（0.887，0.941）	0.700（0.613，0.781）	0.962（0.941，0.980）	0.901（0.876，0.926）	0.766
	with SCAR-Net	0.978（0.943，0.996）	0.971（0.954，0.987）	0.890（0.825，0.945）	0.995（0.987，0.998）	0.973（0.958，0.985）	0.932
S3	Independent	0.791（0.700，0.869）	0.909（0.880，0.935）	0.673（0.584，0.756）	0.949（0.923，0.968）	0.887（0.857，0.912）	0.727
	with Swin-Unet	0.879（0.815，0.943）	0.906（0.876，0.935）	0.690（0.602，0.776）	0.969（0.951，0.986）	0.901（0.872，0.929）	0.773
	with SCAR-Net	0.967（0.928，0.997）	0.966（0.947，0.984）	0.871（0.806，0.935）	0.992（0.982，0.997）	0.966（0.950，0.983）	0.917

Table S8. MRMC analysis comparing independent and AI-assisted diagnosis across six readers. Related to Figure 4.

Reader group	Condition	Mean AUC (95% CI)	ΔAUC vs. baseline	*p*-value
**Junior (n = 3)**	Independent	0.794 (0.765–0.820)	–	–
	AI-assisted	0.928 (0.905–0.948)	0.134	< 0.001
**Senior (n = 3)**	Independent	0.838 (0.812–0.861)	–	–
	AI-assisted	0.954 (0.937–0.968)	0.116	< 0.001
**All readers (n = 6)**	Independent	0.817 (0.796–0.837)	–	–
	AI-assisted	0.941 (0.926–0.954)	0.124	< 0.001

Table S9. Radiologist scoring of SCAR-Net outputs in AI-assisted diagnostic changes. Related to Figure 5.

**Categories**	**Segmentation score (Average, SD)**	**Feature heatmap score (Average, SD)**
**Validation set, *p* values**	$p < 0.001$	$p = 0.385$
Correctly altered cases	3.82 ± 0.91	3.75 ± 0.88
Wrongly altered cases	2.39 ± 1.03	3.62 ± 0.96
**External test set, *p* values**	$p < 0.001$	$p = 0.341$
Correctly altered cases	3.78 ± 0.94	3.81 ± 0.86
Wrongly altered cases	2.51 ± 0.99	3.68 ± 0.93

Table S10. Statistics on disagreement cases and diagnostic changes in the validation set. Related to Figure 5.

Radiologist	Disagreement cases	Correctly altered	Wrongly altered	No diagnostic changes
J1 Recurrence	18	15	0	3
J1 Scar	12	9	1	2
J2 Recurrence	19	17	1	1
J2 Scar	33	28	2	3
J3 Recurrence	11	10	0	1
J3 Scar	15	11	1	3
S1 Recurrence	15	13	0	2
S1 Scar	15	10	0	5
S2 Recurrence	14	11	1	2
S2 Scar	34	27	1	6
S3 Recurrence	11	8	0	3
S3 Scar	22	14	1	7
Average Junior	36	30	1.7	4.3
Recurrence	15.9	14	0.3	1.6
Scar	20	16	1.3	2.7
Average Senior	37.1	27.7	1	8.4
Recurrence	18.7	15.7	1	2
Scar	23.7	17	0.7	6
Overall Average	36.4	28.8	1.3	6.3
Recurrence	14.6	12.3	0.3	2
Scar	21.8	16.5	1	4.3

Table S11. Statistics on disagreement cases and diagnostic changes in the external test set. Related to Figure 5.

Radiologist	Disagreement cases	Correctly altered	Wrongly altered	No diagnostic changes
J1 Recurrence	19	15	1	3
J1 Scar	60	51	2	7
J2 Recurrence	22	17	1	4
J2 Scar	37	30	0	7
J3 Recurrence	18	12	0	6
J3 Scar	43	36	1	6
S1 Recurrence	19	15	1	3
S1 Scar	16	8	1	7
S2 Recurrence	22	16	1	5
S2 Scar	46	36	0	10
S3 Recurrence	23	17	1	5
S3 Scar	33	22	0	11
Average Junior	66.4	53.7	1.7	11
Recurrence	19.7	14.7	0.7	4.3
Scar	46.7	39	1	6.7
Average Senior	53	38	1.3	13.7
Recurrence	38.4	31	0.7	6.7
Scar	31.7	22	0.3	9.4
Overall Average	59.6	45.8	1.5	12.3
Recurrence	20.4	15.3	0.8	4.3
Scar	39.2	30.5	0.7	8
